# Brain-Derived Neurotrophic Factor Secreting Human Mesenchymal Stem Cells Improve Outcomes in Rett Syndrome Mouse Models

**DOI:** 10.3389/fnins.2021.725398

**Published:** 2021-10-06

**Authors:** Hyo Jeong Kim, Delger Bayarsaikhan, Jaesuk Lee, Govigerel Bayarsaikhan, Bonghee Lee

**Affiliations:** ^1^Department of Anatomy & Cell Biology, Gachon University College of Medicine, Incheon, South Korea; ^2^Department of Pediatrics, Gil Medical Center, Gachon University College of Medicine, Incheon, South Korea; ^3^Center for Genomics and Proteomics and Stem Cell Core Facility, Lee Gil Ya Cancer and Diabetes Institute, Gachon University, Incheon, South Korea; ^4^Department of Chemistry, College of Natural Science, Seoul National University, Seoul, South Korea

**Keywords:** Rett syndrome, *MECP2*, brain-derived neurotrophic factor, CRISPR-Cas system, transplantation, mesenchymal stem cell

## Abstract

Rett syndrome (RTT) is a severe X-linked dominant neurodevelopmental disorder caused by mutations in the methyl-CpG-binding protein 2 (*MECP2*) gene; *MeCP2* regulates the expression of brain-derived neurotrophic factor (*BDNF*) and increasing BDNF levels ameliorates RTT symptoms. However, the clinical application of BDNF is limited, because of its short half-life and low penetrance across the blood-brain barrier. In this study, we generated BDNF-secreting mesenchymal stem cells (MSCs) from the human umbilical cord cells, using CRISPR-Cas9. We studied the effects of BDNF-MSCs in *MECP2* knockout and *MECP2*-deficient mice. BDNF-MSCs upregulated the expression of BDNF, pAKT, and pERK1/2 and downregulated that of pp38, both *in vitro* and *in vivo*. In our *in vivo* experiments, BDNF-MSCs increased the body and brain weights in mice. BDNF-MSCs increased the neuronal cell numbers in the hippocampus, cortex, and striatum; in addition, they increased the number of synapses. BDNF-MSCs upregulated BDNF and the activity of BDNF downstream effectors, such as pAKT and pERK 1/2; this upregulation was persistent. In conclusion, BDNF-MSCs generated using CRISPR-Cas9 could be a therapeutic strategy for treating RTT.

## Introduction

Rett syndrome (RTT) is a severe X-linked dominant neurodevelopmental disorder that affects approximately 1 in 10,000 female infants. It is categorized into classical and atypical RTT. Majority (95%) of the cases of classical type RTT are caused by *de novo* mutations in the *MECP2* gene located on the X-chromosome (Pejhan and Rastegar, 2021). Female RTT patients develop normally during the first 6 months of life, but thereafter show regression in motor skills, language, and cognition. Characteristics of RTT include slower growth in head circumference, stereotypic hand movements, and loss of purposeful hand skills. Seizures, ataxia, and breathing disturbances are also common (Percy et al., 2010). *MECP2* gene mutations affect male more severely than female, leading to early fetal death or severe neonatal encephalopathy (Villard, 2007). *MECP2* plays a crucial role in both activation and inhibition/silencing of genes. Brain-derived neurotrophic factor (*BDNF*) is an established target gene of *MECP2*, and the correlation between these two genes in RTT is widely studied. Deficiency of *MECP2*, through phosphorylation of the *MECP2* gene or epigenetic modifications, leads to a reduction in the expression, translation, and stability of *in vivo* BDNF (Damen and Heumann, 2013). Animal models of RTT show that BDNF is downregulated in the brain, serum, and cerebrospinal fluid; BDNF deficiency leads to symptoms and outcomes similar to that of *de novo* mutations in the *MECP2* gene. Overexpression of BDNF against MECP2 deficiency in RTT model mice suggests that BDNF could be an alternative therapeutic for RTT (Vanhala et al., 1998; Riikonen, 2003; Abuhatzira et al., 2007).

There is no cure for RTT; however, results from preclinical studies suggest a wide range of therapeutic strategies for RTT, including direct administration of supplements such as MECP2 and BDNF to relieve symptoms (Luoni et al., 2020; Collins et al., 2021). Drugs that increase the levels of BDNF such as glatiramer acetate, known as a multiple sclerosis immune modulator, could be used to recover neuronal function by stimulating the serotonin receptor 7 in conjunction with inhibiting PTP1B, which blocks the TRKB receptor of BDNF (Krishnan et al., 2015; Napoletani et al., 2021; Vigli et al., 2021). However, evaluating their safety and efficacy are critical, because of dosage-sensitive secondary responses, poor stability, and *in vivo* penetration abilities (Tsai, 2012; Lombardi et al., 2017). Advances in gene engineering technologies, such as TALEN and CRISPR, enable successful and continuous introduction of therapeutic molecules with a short half-life, for alleviating the pathogenesis of genetic disorders (Bayarsaikhan et al., 2020; Lee et al., 2020).

We hypothesized that a combination of therapeutic strategies, such as targeted PTP1B injection followed by continuous overexpression of BDNF via local injection of CRISPR/Cas9 - edited MSCs could be a potential strategy for managing RTT. We believe that this would significantly eliminate the limitations in the clinical translation of previously developed methods. To overcome the limitations of BDNF administration, we generated BDNF-secreting mesenchymal stem cells (MSCs) from the human umbilical cord (UC) cells using the CRISPR/Cas9 system. A continuous supply of BDNF via the secretion from MSCs could ameliorate the symptoms of RTT by restoring neuronal size, morphology, and synaptic function. This study investigated whether BDNF-secreting MSCs generated by CRISPR/Cas9 could reverse the neuronal pathology in MECP2-deficient RTT mouse models and in neurons that underwent *MECP2* knockdown. We also aimed to identify the downstream mechanisms of MeCP2 and BDNF.

## Materials and Methods

### Cell Culture

Umbilical cord blood-driven MSCs (UCB-MSCs) (Medipost) were grown in α-MEM medium (DMEM, Gibco) with 10% fetal bovine serum (FBS, Gibco) and 1% penicillin streptomycin (Sigma-Aldrich). The cells were kept in an incubator under humidified conditions at 37°C and 5% CO_2_. The culture medium was changed three times per week, and cells were sub-cultured at 80% confluence.

### Generation of Brain-Derived Neurotrophic Factor-Secreting Umbilical Cord Blood-Driven-Mesenchymal Stem Cells

To generate BDNF-secreting UCB-MSCs, transfection was performed using the CRISPR/Cas9 ribonucleoprotein system (ToolGen, Inc.), which targets a safe harbor site known as adeno-associated virus site 1 (AAVS1). The donor vector was purchased from Sigma Aldrich (pZDonor-AAVS1 Puromycin vector, PZD0020), and the FLAG-tagged human BDNF encoding sequence was cloned into the multiple cloning site. Nucleofection was performed using a 4D nucleofector (Lonza) by transfecting 15 μg of Cas9 protein, 20 μg of gRNA (GGGGCCACTAGGGACAGGAT), and 1 μg of donor DNA into 8 × 10^5^ UCB-MSCs. Transfected cells were incubated at 37°C for 3 days to stabilize cell conditions. On day 4, the cells were treated with 1 μg/ml puromycin for 5 days to select the positive cells (with successful genome integration of the target sequence). Media was changed once in every 2 days and the culture was maintained until cell confluence was reached and it was ready for injection.

### MeCP2 Gene Silencing by siRNA

SHSY-5Y neuroblastoma cells were seeded at a density of 5 × 10^5^ cells per well of a 6-well culture dish. After 24 h, cells were transfected with MeCP2 siRNA (QIAGEN, HS_MeCP2_7, SI02664893, target sequence ACGGAGCGGATTGCAAAGCAA) at final concentrations of 5 and 200 nM using the FuGene-6 transfection reagent (Roche), according to the manufacturer’s instructions. Forty-eight hours after transfection, cells were harvested, and RNA was isolated using the RNAesy mini kit (QIAGEN; 74104). MeCP2 expression was quantified using RT-PCR.

### Rett Syndrome Mouse Model

The animal experiments were performed at the Center of Animal Care and Use (CACU), Lee Gil Ya Cancer and Diabetes Institute of GACHON University. Five-week-old *MECP2*-deficient male mice (Mecp2^–/y^) (The Jackson Laboratory, stock no: 003890, *Mecp2*^TM 1^.^1Bird^) and 5-week-old male C57BL/6N mice were randomly divided and housed with five mice per cage in a temperature-controlled environment with a 12 h light/dark cycle. The mice had free access to food and water. All animal protocols described in this study were approved by the CACU Animal Center Ethics Board (LCDI-2018-008).

### Stereotaxic Surgery and Tissue Preparation

When *MECP2*-deficient mice were 6 weeks old, the animals were divided into three groups: RTT, BDNF-protein-injected, and BDNF-secreting UCB-MSC-injected groups. In addition, the PTP1b inhibitor CPT157633 (Sigma-Aldrich, 539741) was intraperitoneally injected into the rhBDNF/BDNF-MSC injected mice every other day at a dose of 5 mg/kg to activate the BDNF receptor (TRKB) prior to the 2-week period of cell transplantation experiments. At 8 weeks of age, the RTT model animals were sacrificed (Krishnan et al., 2015). Animals were anesthetized using isoflurane prior to the surgical procedures. A lateral injection was performed into the left ventricle of the brain (anterior: 0.3 mm, lateral: 1.0 mm, dorsal: 3.0 mm from Bregma). For protein injection, 500 ng of BDNF protein (Abcam, ab9794) was prepared in 10 μL of saline. For the UCB-MSC injection, 5 × 10^5^ cells were prepared in 10 μl of α-MEM medium without FBS and antibiotics. Perfusion was performed under anesthesia by injecting the heart with 50 mL of 1 × PBS followed by 50 mL of cold fixative consisting of 4% paraformaldehyde (PFA).

### Enzyme-Linked Immunosorbent Assay

The total BDNF secreted in the cell culture medium was quantified using the Human BDNF Simple Step ELISA kit (Abcam; ab212166), according to the manufacturer’s instructions.

### Western Blot Analysis

Brain tissue and total cell lysates were prepared for western blotting by adding the EzRIPA lysis buffer (ATTO; WSE-7420) and 1 × protease/phosphatase inhibitor (ATTO; WSE-7420), followed by sonication. The tissue lysate was centrifuged at 14,000 × *g* for 20 min at 4°C, and the supernatant was collected. Total protein concentration was measured using the BCA protein assay (Life Technologies; 23227), according to the manufacturer’s instructions. Equal amounts (20 μg) of protein were electrophoresed on 10% polyacrylamide gels (Life Technologies; WG1203BOX) and transferred to a PVDF membrane (Millipore; GSWP01300). Proteins were detected using protein-specific antibodies (Table 1); ECL detection reagent (Life Technologies; 32109) was used to visualize the immunoreactive proteins on the membrane.

**TABLE 1:** List of primer used in this study.

Gene	Direction	Sequence	Tm	Size
GAPDH	Forward	ACCCAGAAGACTGTGGATGG	59°C	415 bp
GAPDH	Reverse	TGCTGTAGCCAAATTCGTTG	55°C	415 bp
MeCP2	Forward	CAGGTCATGGTGATCAAACG	56°C	278 bp
MeCP2	Reverse	AGTCCTTTCCCGCTCTTCTC	59°C	278 bp

### Polymerase Chain Reaction

RT-PCR was performed to determine the expression levels of target genes. Total RNA was isolated using RNAesy mini kit (QIAGEN; 74104) from the total cell lysate, according to the manufacturer’s instructions. RNA was converted to cDNA using a cDNA synthesis kit (Takara, 6110A) and used for RT-PCR experiments with target-specific primers (Table 1).

### Immunostaining

Mouse brain tissues were deparaffinized in xylene for 10 min at room temperature and hydrated using a graded ethanol series (100% ethanol for 5 min, 90% ethanol for 5 min, 80% ethanol for 5 min, and 70% ethanol for 5 min). Briefly, the remaining ethanol was washed with distilled water. Before the incubation with antibody, tissues were heated in antigen retrieval buffer and washed in 1× PBS for five times.

The tissue samples were incubated with primary antibodies (Table 2) overnight at 4°C. The excess antibodies were removed by washing with 1× PBS thrice, followed by incubation with a secondary fluorescent antibody (Table 1) at room temperature for 1 h. Nuclei were counterstained with DAPI (4′6-diamino-2-phenylindole; 1 μg/ml, Invitrogen, D1306) for 20 s at room temperature. After washing with PBS, cover slides were mounted using Vectashield mounting media (Vector Laboratories, H-1000) and images were analyzed using an LSM 710 confocal microscope (Carl Zeiss).

**TABLE 2:** List of antibodies used in this study.

Antigen	Host	Company (Cat. No)	Application IHC WB	
MeCP2	Rabbit	Cell signaling (3456S)		1:1000
Map2	Chicken	Abcam (ab5392)	1:200	
Synapsin	Rabbit	Abcam (ab274430)	1:200	
Akt	Rabbit	Cell signaling (9272S)		
pAkt	Rabbit	Cell signaling (9271S)		
FLAG	Rabbit	Sigma-aldrich (F7425)		1:1000
p38	Rabbit	Cell signaling (9212L)	1:100	1:1000
pp38	Rabbit	Cell signaling (9211S)	1:100	1:1000
ERK1/2	Rabbit	Cell signaling (9102S)	1:100	1:1000
pERK1/2	Rabbit	Cell signaling (4377S)	1:100	1:1000
B -Actin	Rabbit	Abcam (ab8227)	1:100	1:1000
Peroxidase labeled anti-rabbit IgG	Rabbit	Vector (PI 1000)		1:5000
Alexa Fluor 555 donkey anti-rabbit IgG	Rabbit	Invitrogen (A31572)	1:500	
Alexa Fluor 488 donkey anti-chicken IgG	Donkey	Invitrogen (A11001)	1:500	

### Cresyl Violet Staining

Paraffin-embedded mouse brain tissues were deparaffinized in xylene for 10 min at room temperature and hydrated using a graded ethanol series (100% ethanol for 5 min, 95% ethanol for 5 min, and 70% ethanol for 5 min). The brain tissue was washed with distilled water and was stained with 0.5% cresyl violet acetate (Sigma-Aldrich; 10510-54-0) solution for 8 min and washed using a graded ethanol series (70%, 30 s; 80%, 30 s; 95%, 30 s; 100%, 30 s; and 100%, 5 min). The stained slides were mounted with DPX mounting medium (Sigma-Aldrich; 06522) for microscopic image analysis.

### Densitometry

The intensity of each immunoreactivity band was determined using Image J gel digitizing software.

### Statistical Analysis

All data represent results from at least three independent experiments. All experimental data are presented as mean ± standard deviation (SD). Statistical significance was determined using Student’s *t*-test, and a threshold of *P* ≤ 0.05 was considered significant (*: *p* < 0.05, **: *p* < 0.01, ***: *p* < 0.001).

## Results

### Overexpression of Brain-Derived Neurotrophic Factor in CRISPR/Cas9 Mediated Mesenchymal Stem Cells

**FIGURE 1:**
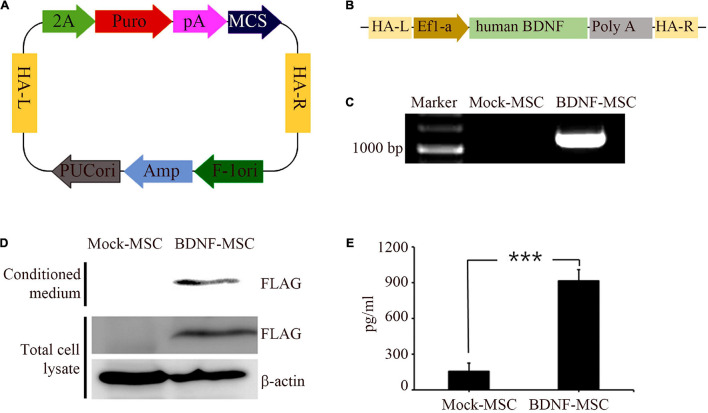
Generation and characterization of BDNF secreting UCB-MSC. **(A)** pZDonor AAVS1 Puromycin vector. **(B)** Cloning of BDNF coding sequences, EF1α promotor, and poly A tail. **(C)** Genome integration confirmed using Junction PCR with genomic DNA of UCB-MSCs, which were transfected with mock and BDNF containing pZDonor-AAVS1 vector. **(D)** Conditioned media and cell lysates from BDNF-secreting UC-MSCs tested using western blotting with FLAG antibodies. Only the transfection-derived BDNF protein can be detected using this method. **(E)** BDNF levels quantified using ELISA with BDNF antibodies. ***p < 0.001.

Immunocytochemistry analysis was performed to validate and distinguish the expression of gene engineered BDNF from that of the naturally occurring proteins using FLAG antibodies (Figures 1A,B). The positive expression of FLAG was observed in both the conditioned medium and the cell lysate of MSCs transfected with pzDonor, but there was no FLAG expression in mock transfected MSCs (Figures 1C,D).

**FIGURE 2:**
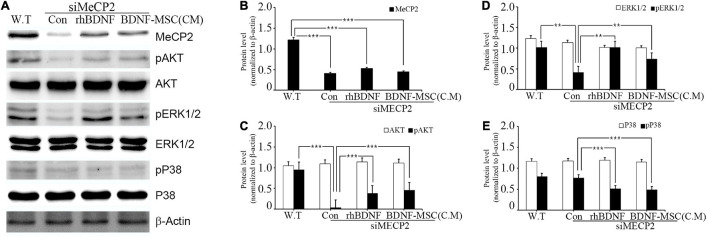
Relative levels of MeCP2, AKT, and MAPK following rhBDNF or BDNF-MSC(CM) treatment in *MeCP2* silenced cells. **(A)** Immunoblot analysis of the *MeCP2* silenced cell lysates following rhBDNF or BDNF-MSC(CM) treatment. **(B–E)** Densitometry analyses of MAPK proteins evaluated using the Image-J software. Conditioned media from the control, si*MeCP2*, BDNF-treated, and BDNF-MSC-treated groups were tested. The phosphorylation of AKT and MAPK (ERK1/2 and p38) indicates changes in cell signaling. β-actin was used as the standard. **p < 0.01, ***p < 0.001.

After validating the overexpression of BDNF in the genetically modified MSCs, we performed ELISA to measure the amount of secreted BDNF. We sampled the conditioned medium of BDNF-transfected MSCs and wild-type MSCs, which were maintained at comparable confluency and culture duration. The BDNF secretion level was 5.85 times higher in the medium with the cells harboring gene edited MSCs, compared to that in the medium with wild-type MSCs (Figure 1E). We detected 914 pg/ml of BDNF in the conditioned medium from BDNF-MSCs and 156 pg/mL (*p* = 0.001; Figure 2B), from that of mock MSCs.

### *In vitro* Assay for Therapeutic Effects of Brain-Derived Neurotrophic Factor-Mesenchymal Stem Cells on Rett Syndrome-Modeled Cell Line

#### Cellular Model for Rett Syndrome

**FIGURE 3:**
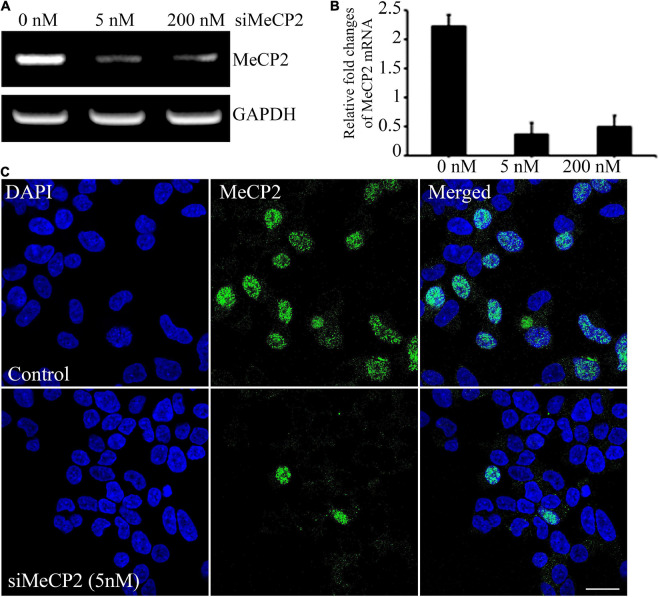
Efficiency of *MeCP2* silencing in SHSY-5Y neurons. **(A)** *MeCP2* expression levels in knockdown cells, assessed using PCR, following treatment with different concentrations of *MeCP2* siRNA. **(B)** Relative changes in expression are presented in a bar graph. **(C)** Immunohistochemistry for assessing the presence and localization of MeCP2. The cells were dual stained for the nucleus (blue) and MeCP2 (green) and observed using confocal microscopy. Scale bar = 50 μm.

To evaluate the therapeutic effect of BDNF overexpressing MSCs, we generated a cellular model of RTT through siRNA-mediated silencing of the *MECP2* in the SHSY-5Y cell line. For silencing the *MECP2* in the neuroblastoma cells, the efficacy of the siRNA at two different concentrations, 5 and 200 nM, were assessed using PCR analysis and immunohistochemical staining (Figures 3A,B). In PCR analysis, the GAPDH gene was used as the housekeeping gene. Quantitative analysis of *MECP2* protein expression in the immunohistochemical staining was performed using ImageJ software. The expression of MECP2 in the neuroblastoma cells decreased by 6- and 4.5-fold following transfection with 5 and 200 nM of si*MeCP2*, respectively. The lower dose was more efficient than the high dose of siRNA, without severely harming the cells; therefore, for further experiments, we used 5 nM of siRNA. The co-localization of DAPI and MeCP2 was remarkably reduced in si*MECP2* treated cells, compared to that in wild type cells, indicating the successful silencing of the *MECP2* gene in siRNA-transfected cells (Figure 3C).

#### Effects of Brain-Derived Neurotrophic Factor Overexpression

The specific affinity of BDNF toward tropomyosin-related kinase B (TRKB) leads to the downregulation of numerous pathways, such as mitogen-activated protein kinase (MAPK) and phosphatidyl-inositol 3-kinase (PI3K)/protein kinase B (AKT), which promote cell growth, differentiation, survival, and synaptic plasticity of the cell. Therefore, we aimed to investigate whether BDNF expression influenced the downregulation of these pathways. In addition, we examined the influence of BDNF expression on the survival of *MECP2* silenced neuroblastoma cells. Wild type SHSY-5Y was used as the control for the RTT cellular model, in which the *MECP2* gene was silenced. They were treated using either rhBDNF or the conditioned medium of BDNF overexpressing MSCs. The BDNF levels in the conditioned medium was determined using ELISA and, an equal amount of rhBDNF was applied. Western blot analysis demonstrated that the expression of *MeCP2*, *pAKT*, *pERK 1/2*, and *pp38* was downregulated in the *MeCP2* knockdown cells, compared to that in the control cells (Figure 2A). The BDNF-treated and BDNF-MSC-treated groups were treated with a PTP1B (protein tyrosine phosphatase 1 B) inhibitor. PTP1B targets the TRKB receptor and functions as a negative regulator of BDNF signaling. Therefore, we applied a PTP1B inhibitor to prevent the weakening of BDNF signaling. Both BDNF-treated and BDNF-MSC-treated groups had increased levels of pAKT and pERK 1/2 and decreased levels of pp38, compared to that in control (Figures 2B–E).

### *In vivo* Testing of Therapeutic Effects of Brain-Derived Neurotrophic Factor Overexpression via Mesenchymal Stem Cells in Rett Syndrome

#### Brain-Derived Neurotrophic Factor-Mesenchymal Stem Cells Prevent Loss of Body Weight and Brain Weight in *MECP2* Knockout Mice

**FIGURE 4:**
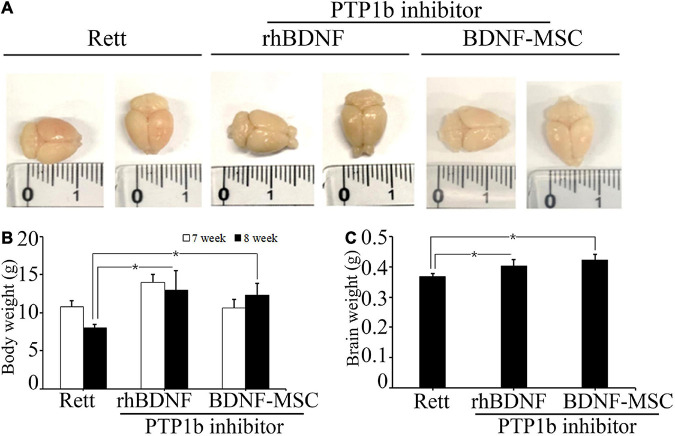
Comparison of body and brain weights. **(A)** Stereotaxic surgery was performed at 7 weeks and brain samples were collected at 8 weeks of age. Brain size was measured after fixation with 4% PFA. **(B)** The average body weights of the animals at 7 and 8 weeks of age. **(C)** Brain weight represented in a bar graph. *p < 0.05.

We evaluated the therapeutic effects of BDNF secreting MSCs in RTT animal models at the tissue level. The *MECP2* knockout mice models were transplanted with BDNF-overexpressing MSCs at an animal age of 6 weeks via surgical procedures and their body weight and brain weights were measured (Figure 4A). *MECP2*-deficient mice gradually lost weight and experienced frequent ataxia over time. There was no loss of body weight in the BDNF-MSC-treated groups, and there was statistically significant difference between the saline injected group and the BDNF-MSC-treated group after 2-weeks of therapy (*p* = 0.048) (Figure 4B). The mice were sacrificed 2 weeks after injecting either rhBDNF or BDNF overexpressing MSCs and their brain size and brain weight were measured. Among the three groups of RTT model mice, which were injected with saline solution, rhBDNF, or BDNF overexpressing MSCs, the control group showed the smallest brain size and brain weight, while the largest brain size and brain weight was found in the BDNF-MSC-treated groups (Figure 4C). Therefore, intraperitoneal application of BDNF promoted cell survival and enhanced the regeneration of brain tissue. The constitutive *in vivo* secretion in the brain ventricle prevented the loss of brain weight and size in *MECP2* knockout mouse models more efficiently, when compared to that using the synthetic form of the protein.

### Brain-Derived Neurotrophic Factor-Mesenchymal Stem Cells Promotes Growth of Neuronal Cells in the *MECP2* Knockout Mice

**FIGURE 5:**
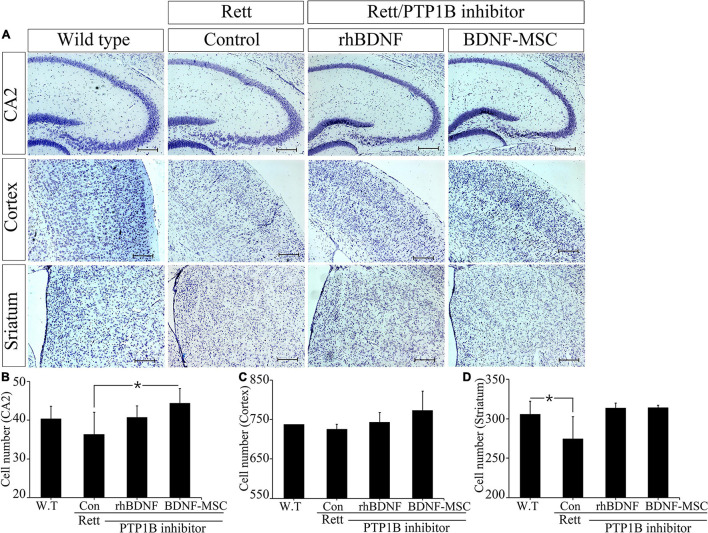
Improved neurogenesis following BDNF-MSC treatment. **(A)** The samples from wild type, *MECP2* knockout (Control-Rett), *MECP2* knockout co-treated with BDNF/PTP1b inhibitor or BDNF-MSC/PTP1b groups were stained with cresyl violet. The cell numbers for each group were counted in three different areas of the brain: **(B)** CA2 of the hippocampus, **(C)** cortex, and **(D)** striatum. Cell numbers were counted using the ImageJ software and they are presented in the graphs. *p < 0.05.

We narrowed the focus to the cellular level to understand how the brain weight and size were influenced by treatment with BDNF in the RTT model mice. Tissues sampled from the CA2 of the hippocampus, cortex, and striatum of the experimental animals from the four groups, including wild type mice (control) and *MECP2* knockout mice treated with saline, rhBDNF, or BDNF-MSCs were stained with cresyl violet (Figure 5). Analysis using Image J software demonstrated that there was significant difference between the number of neurons in the CA2 of hippocampus of wild type and *MECP2* deficient mice (*p* = 0.001). The number of neurons in the hippocampus of wild type mice and *MECP2* knockout mice were 38.7 ± 5.5 and 36.3 ± 5.7, respectively, at 8 weeks of age. After transplantation of rhBDNF-or BDNF-secreting MSCs into RTT model mice, the number of neurons increased to 40.7 ± 3.1 and 44.3 ± 3.8, respectively. These results suggest that RTT induced a reduction in the number of neurons and the neurogenesis in the hippocampus. However, these tend to improve in the presence of BDNF at the age between 6 and 8 weeks in *MECP2* knockout mice. BDNF-MSC treatment was more efficient than the injection of the recombinant form of the protein.

There was no difference in the number of neuronal cells in the cortex, between the control group and RTT group, which was 737.3 ± 7.8 and 725.7 ± 12.2, respectively. However, after injection of either rhBDNF or BDNF overexpressing MSCs, the number of cells gradually increased to 743.3 ± 24.0 and 773.0 ± 49.0, respectively. Therefore, BDNF increased neurogenesis in the cortex, and application of an *in vivo* expression technique for BDNF protein was more efficient in promoting neuronal cell growth in the cortex of *MECP2* deficient mice.

We analyzed neurogenesis in the striatum of the brain samples obtained from experimental animals. The self-renewal capacity of the striatum neurons was relatively less in the *MECP2* deficient mice, compared to that in the wild type mice. Two weeks after rhBDNF and BDNF-MSC injection, the number of neurons were 313.3 ± 6.7 and 314.0 ± 3.0, respectively, and which were higher than that of saline injection group (274.7 ± 28.0). However, wild type mice had an average of 305.7 ± 16.3 striatal neurons. The neurons in the striatum were activated by rhBDNF and BDNF-MSCs, similar to that in the hippocampal and cortical regions.

### Brain-Derived Neurotrophic Factor-Mesenchymal Stem Cells Treatment Increased Synapses in *MECP2* Knockout Mice

**FIGURE 6:**
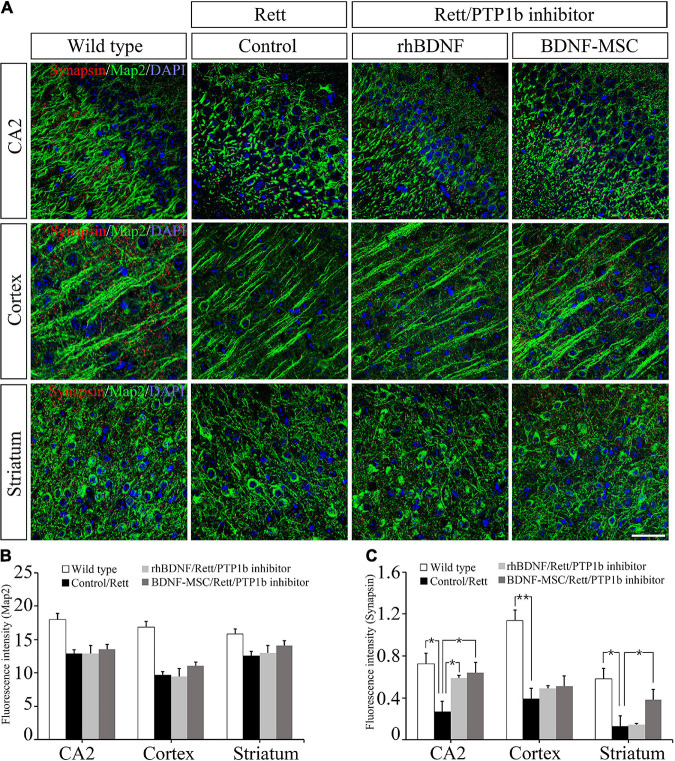
Immunostaining analysis for detection of synapses. **(A)** Immunostaining confocal image showing expression of Map2 (green), synapses (red), and DAPI (blue) in mouse brain tissue. The expression intensity of **(B)** Map2 and **(C)** synapses were measured using ImageJ software. Scale bar = 50 μm *p < 0.05, **p < 0.01.

Two weeks post-injection of BDNF/BDNF-MSCs, immunostaining was performed on mouse brain tissues sampled from CA2 of the hippocampus, cortex, and striatum. Dysfunction of the X-linked *MECP2* gene leads to diminished signaling in the junctions between the neurons located in the CA2 hippocampus, cortex, and striatum regions of the brain (Figure 6A). There were no considerable differences in the neurons responding to the MAP2 marker, among the *MECP2* knockout mice injected with saline, rhBDNF, and BDNF-MSCs; however, they showed considerable reductions when compared to that in the wild type mice (Figure 6B).

We evaluated whether the presence of BDNF and deficiency of PTP1B affects the synaptic abilities of whole neurons located at different distances from the initial injection site in the brain. BDNF expressing MSCs highly altered the synaptic abilities of striatal neurons, and there were alterations in the CA2 hippocampal and cortical neurons, which were proportional to the distance from the injection site (Figure 6C).

### Brain-Derived Neurotrophic Factor-Mesenchymal Stem Cells Treatment Increased pAKT and pERK 1/2 and Decreased pp38 Expression in *MECP2* Knockout Mice

**FIGURE 7:**
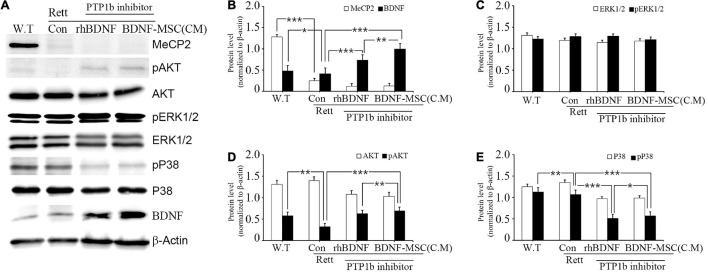
Relative levels of BDNF, AKT, and MAPK following BDNF or BDNF-MSC treatment in *MECP2* knockout mice. The activity of the downstream signaling pathways of BDNF in mice was determined using western blotting **(A)**. Phosphorylation of MAPK represents a change in the activities of cell signaling pathways. **(B–E)** Relative intensity is represented in graphs. *p < 0.05, **p < 0.01, ***p < 0.001.

We investigated the relationship between the expression of BDNF and its downstream signaling pathways in *MECP2* knockout mice using western blotting. There was no expression of MECP2 in the RTT model mice, while wild type mice had a high expression of MECP2 (Figure 7A). Analysis of the expression of total and phosphorylated forms of AKT, ERK1/2, and p38 indicated that phosphorylation of AKT and ERK1/2 increased and that of p38 decreased with the inhibition of PTP1B gene and the transplantation of BDNF protein in *MECP2* knockout mice, compared to that in the wild type or *MECP2* knockout mice injected with saline (Figures 7A–E). The comparison between groups treated with rhBDNF and BDNF-MSCs showed that their phosphorylation effects were similar for pERK1/2 (Figure 7C). However, transplantation of BDNF-MSCs resulted in a higher phosphorylation of pAKT and a decreased expression of pP38 (Figures 7D,E). These results suggest that a continuous *in vivo* expression of BDNF could have better therapeutic effects, for improving the growth and functional activity of neurons during the pathogenesis of RTT.

## Discussion

We cloned a 2.1 kb insert containing the BDNF gene into the AAVS1 site of UC-MSCs, using the CRISPR/Cas9 system. Gene engineering could increase the secretion of the BDNF by at least 5.85-folds, compared to that in wild type cells. BDNF augmentation could be performed using various carriers, including viruses and nanoparticles (Gao et al., 2016; Lopes et al., 2017). This study introduced a more efficient and safer carrier, the MSCs. Delivering BDNF via MSCs overcomes the limitations of poor diffusion into the blood-brain barrier and short half-life, when compared to the use of recombinant BDNF (Khalin et al., 2016). Inactivation of the TRKB receptor results in a gradual decrease in the BDNF levels in the entire brain during the pathogenesis of RTT. In mammals, BDNF plays a crucial role in the survival, growth, and differentiation of neuronal populations (Numakawa et al., 2010). Therefore, in a therapeutic perspective, effective overexpression of such essential neurotrophic factors is the key to curing the disease without causing severe and prolonged pathogenesis. Increased passage of MSCs naturally boost secretion of BDNF; MSCs are promising delivery systems for therapeutics in various neurodegenerative disorders, such as Alzheimer’s disease and Parkinson’s disease, because of their secretomes (Rodrigues Hell et al., 2009; Harrell et al., 2021; Jalali et al., 2021; Kuo et al., 2021). Therefore, BDNF-MSCs could be effective in treating RTT, because of their ability to overexpress BDNF and the presence of neuroprotective or immunomodulatory exosomes.

To investigate the therapeutic effects of BDNF/BDNF-MSCs on RTT, we performed parallel experiments on *MECP2*-silenced neuroblastoma cells (SHSY-5Y) and *MECP2* deficient mice. In both experiments, we injected BDNF-MSCs in conjunction with a PTP1B inhibitor. PTP1B is a major metabolic regulator that attenuates insulin and leptin signaling. PTP1B levels are increased in mouse models of RTT (Krishnan et al., 2015; Fukuhara et al., 2019; Köhn, 2020). PTP1B targets tropomyosin-related kinase B (TRKB; the receptor for BDNF) and negatively regulates BDNF signaling (Ozek et al., 2014). Therefore, we used a PTP1B inhibitor to prevent the loss of BDNF signaling. To further investigate the mechanisms underlying the enhancement of neuronal populations, the effects of BDNF on the activation of AKT and ERK1/2 survival signaling and p38 apoptosis signaling, were evaluated, with respect to the expression levels of BDNF and MECP2. Both *in vivo* and *in vitro* studies using the PTP1B inhibitor showed that BDNF/BDNF-MSCs induced the activation of TRKB; the upstream signaling pathways, including ERK1/2 and AKT, were activated. However, the p38 pathway, which is a signaling pathway downstream of the interaction between TRKB and BDNF, was inhibited. There were significant differences among the expression levels of these upstream and downstream cascades, between saline-injected and BDNF/BDNF-MSC-injected groups, indicating that BDNF activates the TRKB receptor after blocking PTP1B. Among the studied cascades, ERK1/2 is known for its role in the expression of synaptic proteins and RTT is considered a synaptic development disorder caused by the dysfunction of *MECP2* (Johnston et al., 2003; Kumamaru et al., 2011). Recent animal study shown that TRKB plays crucial role in restoration of hippocampal synaptic plasticity in MECP2 mutant mice (Li et al., 2017). The findings in the present study also supported this finding, and it was observed that local injection of rhBDNF/BDNF-MSCs gradually increased neuronal numbers and cell synapses. Considerable improvements in synaptic plasticity and signaling were observed in CA2 of the hippocampus and striatum, while minor changes were observed in the cortex in the *MECP2* knockout mice, in response to BDNF treatments. The difference in response could be explained by the distance from the initial injection site in the ventricle and the distribution rate of the injected therapeutic molecules. The striatum is the closest to the ventricle and the highest increases in the neural synapse was observed in this area. The strength of the synapse decreased in the hippocampus and cortex, which were farther from the injection site. In addition, cresyl violet staining indicated similar improvements in neurogenesis. The BDNF-injected groups exhibited increased neurogenesis, compared to the saline-injected group. The increase in cell numbers in the striatum was similar between the rhBDNF and BDNF-MSCs injected groups. However, the cell number significantly increased in the cortex and hippocampus of the BDNF-MSC-injected group. This could be attributed to the short half-life of BDNF. The increase in neuronal population was in the following series according to the distance from the initial injection point (ventricle): striatum > hippocampus > cortex in regard with treatment of BDNF/BDNF-MSC. The body weights of the mice were measured at the first and second week following BDNF/BDNF-MSC treatment and there were losses observed in the groups treated with saline and rhBDNF injections. However, the administration of BDNF-MSCs resulted in weight gain. In addition, the highest size and weight of the brain was found in the BDNF-MSC-injected group, followed by the groups administered with rhBDNF and saline.

## Conclusion

In conclusion, the local injection of rhBDNF or BDNF-MSCs in conjunction with the inhibition of PTP1B in *MECP2* deficient mice could lead to increase neurogenesis and syanpses through BDNF-dependent mechanisms such as ppAKT and ppERK 1/2. This led to increased brain weight, reduced loss in body weight and improved survival in RTT mice. Althogh, further studies are required for ascertaining the safe and effective dosage of BDNF-MSC and methods for delivering BDNF-MSC, BDNF-MSC treatment is a promising therapeutic strategy for treating RTT.

## Data Availability Statement

The raw data supporting the conclusions of this article will be made available by the authors, without undue reservation.

## Ethics Statement

The animal study was reviewed and approved by Animal Center Ethics Board, Center of Animal Care and Use, Lee Gil Ya Cancer and Diabetes Institute of GACHON University, (LCDI-2018-008).

## Author Contributions

BL contributed to the conception and design of the study. HK and DB performed data analysis and wrote the manuscript. DB, HK, JL, and GB performed the experiments and participated in drafting the manuscript. All authors read and approved the final manuscript.

## Conflict of Interest

The authors declare that the research was conducted in the absence of any commercial or financial relationships that could be construed as a potential conflict of interest.

## Publisher’s Note

All claims expressed in this article are solely those of the authors and do not necessarily represent those of their affiliated organizations, or those of the publisher, the editors and the reviewers. Any product that may be evaluated in this article, or claim that may be made by its manufacturer, is not guaranteed or endorsed by the publisher.
